# Nivolumab plus Brentuximab vedotin +/- bendamustine combination therapy: a safe and effective treatment in pediatric recurrent and refractory classical Hodgkin lymphoma

**DOI:** 10.3389/fimmu.2023.1229558

**Published:** 2023-07-31

**Authors:** Patrick Greve, Auke Beishuizen, Melanie Hagleitner, Jan Loeffen, Margreet Veening, Marianne Boes, Victor Peperzak, Claudius Diez, Friederike Meyer-Wentrup

**Affiliations:** ^1^ Center for Translational Immunology, University Medical Center Utrecht, Utrecht, Netherlands; ^2^ Department of Hemato-oncology, Princess Máxima Center for Pediatric Oncology, Utrecht, Netherlands; ^3^ Department of Pediatrics, University Medical Center Utrecht, Utrecht, Netherlands

**Keywords:** oncology, recurrent/refractory classical Hodgkin lymphoma, checkpoint inhibition, combination immunotherapy, targeted therapy

## Abstract

**Introduction:**

Classical Hodgkin lymphoma (cHL) is the most common pediatric lymphoma. Approximately 10% of patients develop refractory or recurrent disease. These patients are treated with intensive chemotherapy followed by consolidation with radiotherapy or high-dose chemotherapy and autologous stem cell reinfusion. Although this treatment is effective, it comes at the cost of severe long-term adverse events, such as reduced fertility and an increased risk of secondary cancers. Recently, promising results of inducing remission with the immune checkpoint inhibitor nivolumab (targeting PD-1) and the anti-CD30 antibody-drug conjugate Brentuximab vedotin (BV) +/- bendamustine were published.

**Methods:**

Here we describe a cohort of 10 relapsed and refractory pediatric cHL patients treated with nivolumab + BV +/- bendamustine to induce remission prior to consolidation with standard treatment.

**Results and discussion:**

All patients achieved complete remission prior to consolidation treatment and are in ongoing complete remission with a median follow-up of 25 months (range: 12 to 42 months) after end-of-treatment. Only one adverse event of CTCAE grade 3 or higher due to nivolumab + BV was identified. Based on these results we conclude that immunotherapy with nivolumab + BV +/- bendamustine is an effective and safe treatment to induce remission in pediatric R/R cHL patients prior to standard consolidation treatment. We propose to evaluate this treatment further to study putative long-term toxicity and the possibility to reduce the intensity of consolidation treatment.

## Introduction

Classical Hodgkin lymphoma (cHL) (1) is the most frequent lymphoma in children. Chemo- and radiotherapy results in a 5-year event-free survival (EFS) of 89% (2, 3). However, approximately 10% of patients present with recurrent or refractory (R/R) disease. Currently, there is no uniform standard of care for treatment of pediatric R/R cHL patients. In general, salvage chemotherapy is designed to induce remission with as limited long- and short-term toxicity as possible (4–6). Remission may be induced by IGEV chemotherapy (ifosfamide, gemcitabine, vinorelbine) followed by radiotherapy or HD-BEAM (bendamustine, etoposide, cytarabine and melphalan) chemotherapy with autologous stem cell reinfusion depending on stage of disease at recurrence (4). Achieving a complete metabolic remission (CMR) prior to consolidation therapy predicts outcome of pediatric R/R cHL treatment (7). EFS in adults is better in patients in CMR prior to HD chemotherapy and autologous stem cell reinfusion (8). When choosing the best treatment for remission induction in children with R/R cHL special attention must be paid to efficacy as well as long-term toxicity: Although advances in therapy have reduced the long-term toxicity in cHL survivors, follow-up studies still show increased mortality rates (5.1-fold higher risk of death due to causes other than HL) (9) and late toxicity in this population up to decades after their cHL diagnosis (10, 11). Specifically, premature ovarian failure has been reported in 6-34% of female long-term survivors and elevated FSH levels with low inhibin B in a median of 51.5% and 45% respectively in male survivors (12). In addition, 12.7% of childhood HL survivors develop a novel second cancer within 30 years of the initial diagnosis, which is 11 times higher than the expected incidence in the general population (13). Finally, a 4- to 6-fold increased standardized incidence ratio of coronary heart disease or heart failure compared with the general population has been reported (14).

The malignant HL cells express CD30 and are characterized by upregulated expression of the immune checkpoint molecule programmed death-ligand 1 (PD-L1) that binds to the receptor programmed cell death protein 1 (PD-1) as part of an immune evasive phenotype (15). Consequently, clinical efficacy of anti-PD1 checkpoint inhibition by the anti-PD-1 antibody nivolumab and targeting of malignant cHL cells with the anti-CD30 antibody-drug conjugate Brentuximab vedotin (BV) alone and in combination has been demonstrated in adult (16–19) and partly in pediatric R/R cHL patients (20, 21). Recently, encouraging results of the Checkmate 744 trial were published for children, adolescents and young adults (age 5 to 30 years) with R/R cHL after treatment with Nivolumab + BV +/- bendamustine (22). In this study 31 patients < 18 years were treated. The rate of complete metabolic response (CMR) (assessed according to the Lugano classification) (23) was 59% after induction with nivolumab + BV and 94% before consolidation. The one-year progression free survival rate was reported to be 91%. Nivolumab + BV treatment induced grade 3/4 treatment-related adverse events in 18% of patients. In addition, first results of the treatment for low risk patients consisting of nivolumab + BV followed by radiotherapy have been published recently (24). CMR before radiotherapy was reached in 88.9% of treated children with a 3-y EFS rate of 78.3%.

In the current study we present the real-world experience of treating 10 pediatric R/R cHL patients with nivolumab + BV +/- bendamustine as an effective and safe therapy to induce remission prior to consolidation with HD-BEAM chemotherapy and autologous stem cell reinfusion or radiotherapy.

## Materials and methods

### Patients

We present data on 10 children (<18 years) with R/R cHL treated at the Princess Máxima Center for Pediatric Oncology, Utrecht, The Netherlands. The primary manifestation of cHL was initially treated according to the Euronet PHL-interim or -C2 treatment protocols: 2 OEPA cycles (vincristine, etoposide, prednisolone and doxorubicine) followed by 1 to 4 COPDAC-28 cycles (28 days; cyclophosphamide, vincristine, prednisolone and dacarbazine) or 2 to 4 DECOPDAC-21 cycles (21 days; doxorubicin, etoposide, cyclophosphamide, vincristine, prednisolone and dacarbazine) +/- radiotherapy (25). R/R cHL was confirmed by pathology, when clinically possible, and staging performed by total body MRI scan, FDG-PET-CT scan and abdominal ultrasound. Patients were stratified into 2 groups (standard and low risk) according the EuroNet-PHL guidelines (4) and as published (22, 24). Disease was considered refractory if remission had not been achieved or if progression occurred < 3 months after end-of-treatment. All patients and/or parents or guardians were informed about standard chemotherapy (4) and offered to choose treatment with nivolumab + BV. They provided written informed consent according to national laws and in agreement with the declaration of Helsinki (2013). This study is IRB-approved and registered under a national trial registry number 7744.

### Treatment of R/R cHL

All patients were treated as published for the Checkmate 744 trial (22, 24): Patients received 4-6 cycles of nivolumab (3 mg/kg on day 8 of cycle 1 and on day 1 thereafter) + BV (1.8 mg/kg on day 1 of every cycle). If CMR [Deauville score < 4, according to Lugano criteria (23)] was reached after cycle 4, patients from the low risk group received consolidation treatment by involved-field radiotherapy. After achieving CMR patients from the standard risk group were treated with HD-BEAM chemotherapy followed by autologous stem cell reinfusion. Autologous peripheral CD34+ blood stem cells were harvested in standard risk patients after the 3^rd^ or 4^th^ cycle of nivolumab + BV according to standard procedures. In patients not in CMR after the 4^th^ nivolumab + BV cycle treatment was intensified by administering 2 cycles of BV + bendamustine (90 mg/m2 on day 1 and 2). One patient was treated with DHAP (dexamethasone, high-dose cytarabine, cisplatinum) + BV (26) because of progressive disease after BV + bendamustine. Treatment toxicity was assessed according to the National Cancer Institute Common Terminology Criteria (CTC) to evaluate adverse events.

### Response evaluation

Response to treatment was evaluated by total body MRI scan and FDG-PET-CT after 2 and 4 cycles of nivolumab + BV or after the second cycle of bendamustine + BV according to the Lugano criteria (23). A Deauville score <4 was considered a CMR.

### Laboratory tests

Laboratory tests were performed according to standardized procedures at the laboratories for clinical chemistry at the University Medical Center Utrecht and the Princess Máxima Center. Serum TARC levels were measured by ELISA at the laboratory of the UMC Utrecht.

### Statistical analysis

Data were descriptively analyzed using Stata 18 SE for Windows (StataCorp., College Station, TX, USA). Continuous data were shown as median and range or as mean values and range when useful. Categorical data were shown as frequencies and percentages.

## Results

### Patient characteristics

We here report the results of 10 patients treated for R/R cHL at the Princess Máxima Center for Pediatric Oncology (**Table 1**). The median age at primary cHL diagnosis was 13 years (range: 9-16). Nine children had received first-line cHL therapy according to the EuroNet PHL-C2 treatment protocol and one according to the EuroNet PHL-interim treatment recommendation (25) at our center (**Table 1**). Two patients had received radiotherapy after completion of chemotherapy. Notably, 5 patients were in complete metabolic remission (CMR) at the end of first line treatment.

**Table 1:** Baseline demographics and clinical characteristics at initial diagnosis.

Characteristic	R/R cohort - all patients (n = 10)
Median age (range), years	13 (9 – 16)
< 18 years	10 (100%)
Male sex	7 (70%)
Initial diagnosis (n)	
Classical Hodgkin lymphoma	10
Stage at initial diagnosis	
II III IV	5 (50%) 2 (20%) 3 (30%)
B-symptoms at initial diagnosis	4 (40%)
Original sites of disease (multiple lymph nodes in 1 region not counted separately)	
2 3-5 >5 Lung involvement Bone/marrow involvement	4 (40%) 3 (30%) 3 (30%) 3 (30%) 1 (10%)
Prior systemic therapy	
2×OEPA/2×COPDAC 2×OEPA/2×COPDAC/radiation therapy 2×OEPA/2×DECOPDAC 2×OEPA/4×COPDAC 2×OEPA/4×DECOPDAC/radiation therapy 2×OEPA	3 (30%) 1 (10%) 1 (10%) 3 (30%) 1 (10%) 1 (10%)
Initial response to first line therapy	
CMR/CR PMR/PR Progressive disease	5 (50%) 1 (10%) 4 (40%)
Response status prior to induction therapy	
Refractory Relapsed, 1^st^ relapse Relapsed, 2^nd^ relapse	4 (40%) 5 (50%) 1 (10%)
Median days (range) between diagnosis of first relapse and last day of initial treatment	176 (8 – 804)
Median performance status (range) at recurrence prior to induction therapy Karnofsky	100 (80 – 100)

### Characteristics R/R disease

Recurrences occurred on average 236 days (range 8-804, median 176 days) after end-of-treatment and were diagnosed during routine follow-up visits in 90% of cases. One case came to attention due to a visit scheduled at the patients’ request after feeling a swelling the neck. In 9/10 patients R/R disease was detected in the initially involved lymph node region. 3/10 patients showed progressive disease during treatment (1 at early and 2 at late response assessment). In 20% of cases the relapse occurred at a site previously treated by radiotherapy. One patient presented with a second recurrence. Nine R/R cHL cases were confirmed by pathology. In 1 patient a biopsy was considered too dangerous due to the bleeding risk because of the anatomical localization. The diagnosis of this specific cHL recurrence was made based on detection of enlarging FDG-PET-positive (Deauville score 5) lymph nodes in the previously afflicted lymph node region, an increase of the erythrocyte sedimentation rate (ESR) from normal to 63 mm/1^st^ h and serum TARC-elevation to 971 pg/ml.

In all patients staging was performed with total body MRI and FDG-PET-CT scans. It demonstrated Ann-Arbor stage I/II disease extension in 50% and stage III/IV in 50% of patients. Characteristic “B-symptoms” for cHL such as fever, drenching night sweats or weight loss >10% body weight were absent in 90% of patients treated for R/R disease. One patient with refractory disease had B-symptoms at first presentation. Three patients presented with an ESR >30 mm/1^st^ hour. Recently, the cytokine TARC (CCL-17) has been characterized as a biomarker for cHL in children (27–29). At diagnosis of R/R cHL serum TARC levels were elevated (>880 pg/ml) in 8/10 patients and hemoglobin levels reduced (<7.4 mmol/L) in 4/10 patients.

There were no emergency presentations due to a large mediastinal mass. All patients had a Karnofsky/Lansky score above 80 and presented without relevant comorbidities. There were 8 patients in the standard and 2 patients in the low-risk treatment group (**Table 2**).

**Table 2:** Clinical characteristics of R/R HL patients and response to nivolumab + BV +/- bendamustine.

Patient ID	Risk	R/R Induction	Status after 2x N-BV	Status after 4x N-BV	Status after 2x B-BV	R/R Consolidation	Post consolidation status	CMR follow-up (months)
1	Standard	N-BV + B-BV	PMR	PMR	CMR	RT	CMR	42
2	Standard	N-BV	CMR	CMR	n/a	BEAM	CMR	38
3	Standard	N-BV + B-BV	PMR	PMR	CMR	BEAM	CMR	34
4	Standard	N-BV	CMR	CMR	n/a	BEAM	CMR	35
5	Low	N-BV	CMR	CMR	n/a	RT	CMR	12
6	Standard	N-BV	CMR	CMR	n/a	BEAM	CMR	23
7	Standard	N-BV + B-BV	PMR	PMR	CMR	BEAM	CMR	20
8	Standard	N-BV + B-BV + DHAP-BV (1 cycle)	PMR	PMR	CMR	BEAM + RT	CMR	18
9	Standard	N-BV + B-BV	PMR	PMR	ProgD	BEAM + RT	CMR	12
10	Low	N-BV	CMR	CMR	n/a	RT	CMR	25

N-BV, nivolumab-brentuximab vedotin; B-BV, bendamustine-brentuximab vedotin; PMR, partial metabolic response; CMR, complete metabolic remission; ProgD, progressive disease.

### Remission induction with nivolumab + BV

**Figure 1:**
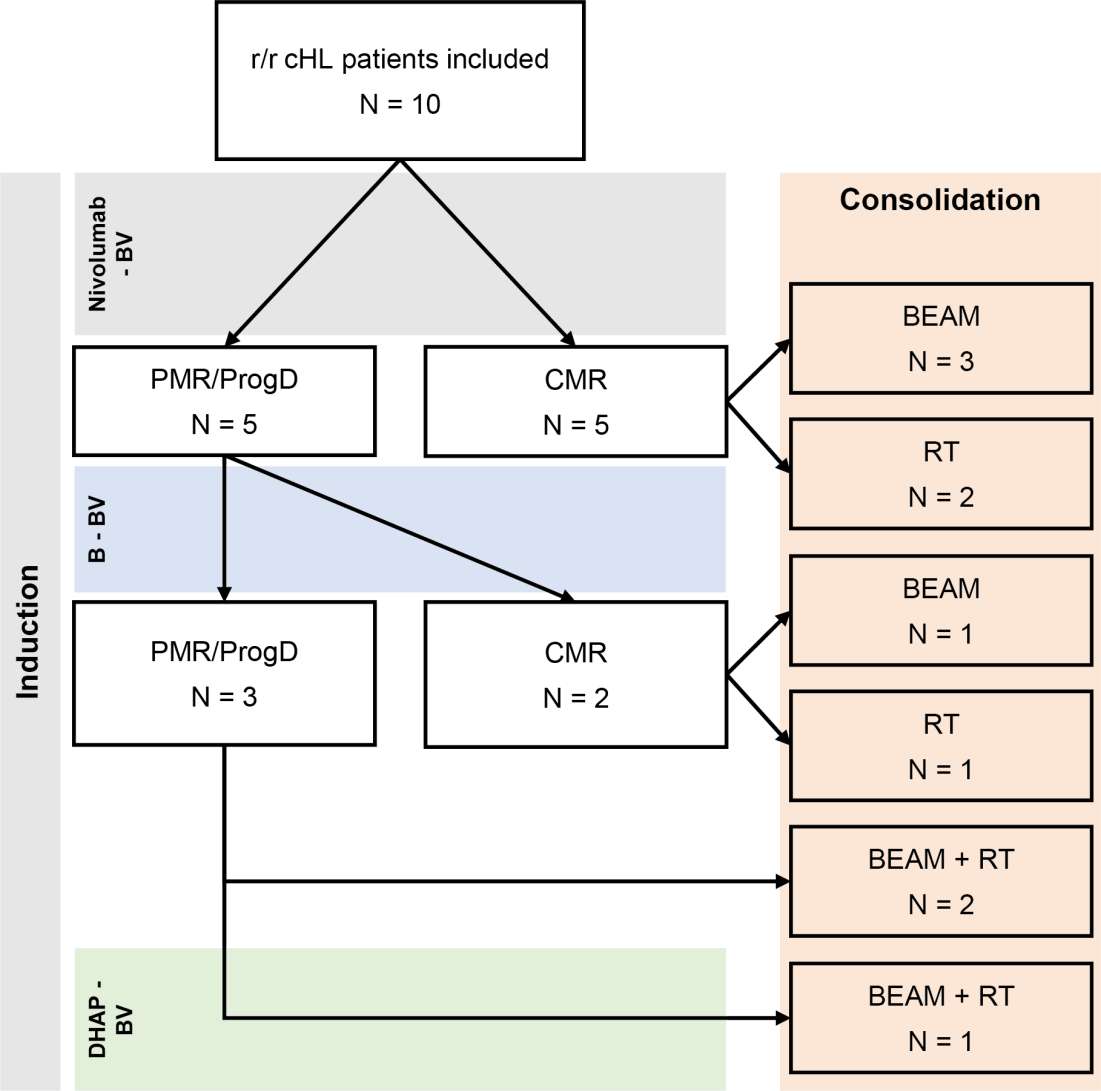
Treatment with nivolumab + BV +/- bendamustine. Flowchart of included patients and their treatment. Five patients had CMR after 4 cycles of N-BV treatment and were consolidated with HD-BEAM (n = 3) or RT (n = 2). Those with PMR continued with 2 cycles of bendamustine + BV, leading to CMR in 4 more patients, consolidated with HD-BEAM (n = 1), RT (n = 1) or HD-BEAM + RT (n = 2). The last patient was treated with a single cycle of DHAP + BV, leading to CMR, and had consolidation therapy with HD-BEAM + RT.

**Figure 2:**
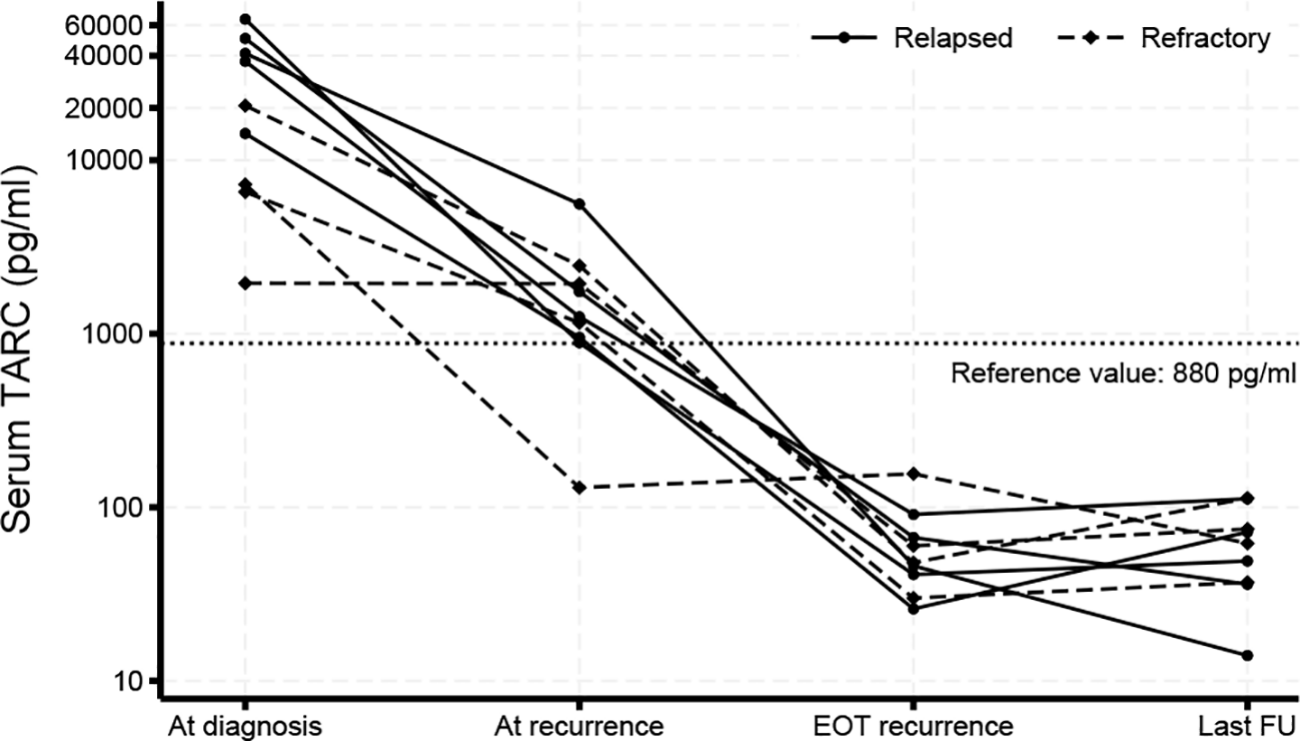
Serum TARC levels. Serum TARC levels were measured by ELISA prior to initiation of treatment of primary disease and at diagnosis of R/R cHL, at end-of-treatment (EOT) of R/R disease and during follow-up (FU). Values above 880 pg/ml were considered elevated based on previous findings (28). TARC levels decreased during treatment and were still normal at the last follow-up moment.

Treatment of R/R disease was on average initiated 16 days (range: 8-26) after diagnosis. Treatment was given according to the Checkmate 744 trial protocol: The first cycle of immunotherapy was started with the administration of BV followed by infusion of nivolumab 7 days later. Afterwards nivolumab + BV was administered on the same day every 3 weeks in our outpatient clinic (22). Immunotherapy induced a CMR (Deauville score <4) in 5 patients after 2 administrations of nivolumab + BV. Four patients did not achieve CMR after 4 nivolumab + BV cycles. Three patients achieved CMR after 2 and 1 patient after 3 cycles of BV in combination with bendamustine. In 1 patient with a FDG-PET-CT scan suspicious for only a partial metabolic response a CT-guided biopsy was performed that did not detect viable cHL tissue. A second patient showed progressive disease after 2 cycles of BV + bendamustine. Treatment was intensified by 1 course of DHAP + BV (26), which induced CMR (**Figure 1**), followed by consolidation with HD-BEAM with autologous stem cell reinfusion. Also this patients is still in ongoing remission for 16 months. TARC levels normalized concomitantly with CMR induction (**Figure 2**). Peripheral stem cell harvest after 2 to 4 cycles of nivolumab + BV was successful in all standard risk patients. On average 5.2 x10^6^ CD34+ cells/kg body weight were harvested.

### Side effects of nivolumab + BV therapy

The treatment was well tolerated and administered in the outpatient clinic (**Table 3**). We observed a single CTC grade 3 or higher adverse event: an increase of ALT and AST more than five times the upper limit of normal. Both ALT and AST recovered spontaneously and there were no signs of impaired liver function and no suspicion of drug-induced liver injury. Bilirubin levels stayed within the reference range. However, the next nivolumab cycle was delayed, while the possible causes were evaluated. Mild side effects that were reported more than once included fever (n = 3), rash (n = 2), and fatigue (n = 2). One patient developed hypothyroidism attributed to nivolumab treatment, which recovered during follow-up.

**Table 3:** CTCAE graded side effects.

Patient ID	CTCAE Term																
	Nausea	Fever	Rash	Treatment delay	Flushing	Headache	Joint effusion	Hypothyr	NCCP	Myalgia	Abdominal pain	Bacteremia	Fatigue	Sneezing	LDH increase	AST increase	ALT increase
**1**																	
**2**	2	1		1												3	3
**3**																	
**4**																	
**5**			1				1										
**6**					1												
**7**								2									
**8**	2	2							2	1	1	2	2	1	1		
**9**		1	1			2											
**10**																	

Adverse events identified in individual patient cases. The value shown in the table corresponds to the CTCAE grade of that specific adverse event. Hypothyr, hypothyroidism; NCCP, non-cardiac chest pain; LDH, lactate dehydrogenase; AST, aspartate transaminase; ALT, alanine transaminase.

### Consolidation treatment

Depending on the initial risk group stratification 7/10 patients received standard consolidation with HD-BEAM chemotherapy plus autologous stem cell reinfusion and 3/10 involved-field RT (30,6 to 36 Gy). One patient with 2^nd^ recurrence had received prior HD-BEAM chemotherapy with autologous stem cell reinfusion as treatment for first recurrence and therefore received radiotherapy as consolation after reaching CMR. In 2/10 patients radiotherapy was administered after completing HD-BEAM chemotherapy with autologous stem cell reinfusion. The indications for additional consolidation radiotherapy in these 2 patients were detection of viable lymphoma after the 4^th^ course of nivolumab + BV with CMR after bendamustine + BV; and progression after treatment with bendamustine + BV with CMR after one cycle of DHAP + BV. Most importantly, these 2 patients achieved CMR before starting consolidation. The toxicity observed after HD-BEAM was mild. No CTCAE adverse events above grade 3 were reported. Grade 3 CTCAE adverse events included febrile neutropenia (n = 5), weight loss (n = 1), nausea (n = 1), vomiting (n = 1), oral mucositis (n = 1) and isolated ALT increase (n = 1). On average 3.8 x10^6^ CD34+ stem cells/kg body weight were infused. Leukocyte numbers had recovered to >5 x10e6/L after a mean of 11 days (range 11-15) after stem cell reinfusion with neutrophils reaching counts >4 x10e6/L after a mean of 13 days (range 10-16). At present all patients are in ongoing remission with a median follow-up time of 25 months (range: 12-42) since the end-of-treatment.

## Discussion

We report that combination immunotherapy with nivolumab + BV (+ bendamustine in 5/10 cases) induces complete remission in 10 pediatric R/R cHL patients and does not cause relevant toxicity during treatment and in the median follow-up period of 25 months (range: 12 to 42). CMR was achieved after only 2 cycles of immunotherapy in 50% of patients. 9/10 patients had achieved CMR after nivolumab +BV +/- bendamustine prior to consolidation treatment. One patient reached CMR after adding DHAP + BV to this treatment. While progressive disease was diagnosed after nivolumab +BV + bendamustine, DHAP + BV induced CMR. This may be due to cHL sensitization to DHAP by prior nivolumab administration (30). All patients are in ongoing remission with a median follow-up period of 25 months. Our findings add real-world evidence to the promising results of the CheckMate 744 study that were recently published (22, 24). Interestingly, nivolumab + BV has also shown promising results in the treatment of R/R primary mediastinal B cell lymphoma (31), a Non-Hodgkin lymphoma entity biologically closely related to cHL (32).

Even though we did not observe relevant toxicity > CTC grade 3, it is important to study possible long-term side effects, most importantly of PD-1 checkpoint inhibition by nivolumab. Children have a more active immune system in development, as characterized by increased T cell responses to infectious agents, when compared to adults, in whom immune senescence increases the presence of dysfunctional and exhausted T cells (33, 34), and could thus be more prone to long-term side effects of checkpoint inhibition. In adults, short- and long-term side effects of checkpoint inhibition are well-characterized and include most prominently different forms of autoimmunity (35). Only 1 possible adverse event related to immunotherapy, hypothyroidism induced by nivolumab, occurred during treatment but resolved spontaneously during follow-up. In the up to 42 months of follow-up we have observed no severe side effects attributable to nivolumab + BV + bendamustine treatment, but longer follow-up is needed to thoroughly compare morbidity and mortality of immunotherapy with current standard of care.

All patients still underwent standard consolidation treatment after reaching CMR. The question remains whether this is necessary. Recently, a case series of 4 children who did not receive HD chemotherapy consolidation after remission induction with BV plus gemcitabine reported ongoing complete response in all of them (36). If patients achieve durable CMR after nivolumab + BV +/- bendamustine without the need for intensive consolidation radio- or chemotherapy, their long-term quality of life may benefit significantly (37, 38).

In our cohort all patients tolerated HD-BEAM chemotherapy followed by autologous stem cell reinfusion very well. Immune reconstitution was fast and patients did not experience symptoms caused by > grade 3 CTC acute toxicity. This could be due to reduced acute toxicity of induction treatment with nivolumab + BV +/- bendamustine when compared to standard chemotherapy (4). This observation could be another benefit of remission induction with nivolumab + BV +/- bendamustine.

In conclusion, nivolumab + BV +/- bendamustine was effective and safe. All patients achieved complete remission before consolidation and are in ongoing complete remission with a median follow-up of 25 months after end-of-treatment. No relevant adverse events of CTCAE grade 3 or higher were reported and no side effects attributable to nivolumab or BV were identified. Our results support that immunotherapy with nivolumab + BV +/- bendamustine is an effective and safe treatment to induce remission in pediatric R/R cHL patients prior to standard consolidation treatment. We propose to evaluate this treatment further to study putative long-term toxicity and the possibility to reduce the intensity of consolidation treatment.

## Data availability statement

The raw data supporting the conclusions of this article will be made available by the authors, without undue reservation.

## Author contributions

PG and AB collected the data and wrote the manuscript, MH, JL, MV, MB, and VP contributed to writing the manuscript, CD analyzed the data and wrote the manuscript and FM-W drafted the manuscript, analyzed data and wrote the manuscript. All authors contributed to the article and approved the submitted version.
